# Antimicrobial Effect of Gentamicin/Heparin and Gentamicin/Citrate Lock Solutions on *Staphylococcus aureus* and *Pseudomonas aeruginosa* Clinical Strains

**DOI:** 10.3390/idr17040098

**Published:** 2025-08-06

**Authors:** Daniel Salas-Treviño, Arantxa N. Rodríguez-Rodríguez, María T. Ramírez-Elizondo, Magaly Padilla-Orozco, Edeer I. Montoya-Hinojosa, Paola Bocanegra-Ibarias, Samantha Flores-Treviño, Adrián Camacho-Ortiz

**Affiliations:** 1 Department of Infectious Diseases, Hospital Universitario Dr. José Eleuterio González, Universidad Autónoma de Nuevo León, Monterrey 64460, Mexico; danielsalast91@gmail.com (D.S.-T.); arannatiluzrod@gmail.com (A.N.R.-R.); tererael@hotmail.com (M.T.R.-E.); e276431@gmail.com (E.I.M.-H.); paola.bocanegraib@gmail.com (P.B.-I.); samflorest@gmail.com (S.F.-T.); 2 Epidemiology Coordination, Hospital Universitario Dr. José Eleuterio González, Universidad Autónoma de Nuevo León, Monterrey 64460, Mexico; magalypadilla2211@gmail.com

**Keywords:** healthcare-associated infections, infection prevention, intravenous devices, bloodstream infection, central venous catheters, decontamination, Gram-negative bacteria, Gram-positive bacteria

## Abstract

**Background/Objectives:** Hemodialysis catheter-related bloodstream infection (HD-CRBSIs) is a main cause of morbidity in hemodialysis. New preventive strategies have emerged, such as using lock solutions with antiseptic or antibiotic capacity. In this study, the antimicrobial effect was analyzed in vitro and with a catheter model of lock solutions of gentamicin (LSG), gentamicin/heparin (LSG/H), and gentamicin/citrate (LSG/C) in clinical and ATCC strains of *Pseudomonas aeruginosa* and *Staphylococcus aureus*. **Methods:** The formation, minimum inhibitory concentration, and minimum inhibitory concentration of the biofilm and minimum biofilm eradication concentration of the lock solutions were determined. Additionally, colony-forming unit assays were performed to evaluate the antimicrobial efficacy of the lock solutions in a hemodialysis catheter inoculation model. **Results:** The minimum inhibitory concentration (MIC) of planktonic cells of both *P. aeruginosa* and *S. aureus* for LSG/H and LSG/C was 4 µg/mL. In the minimum biofilm inhibitory concentration (MBIC) tests, the LSG/H was less effective than LSG/C, requiring higher concentrations for inhibition, contrary to the minimum biofilm eradication concentration (MBEC), where LSG/H was more effective. All lock solutions eradicated *P. aeruginosa* biofilms in the HD catheter model under standard conditions. Nevertheless, under modified conditions, the lock solutions were not as effective versus ATCC and clinical strains of *S. aureus*. **Conclusions:** Our analysis shows that the lock solutions studied managed to eradicate intraluminal mature *P. aeruginosa* in non-tunneled HD catheters under standard conditions. Biofilm inhibition and eradication were observed at low gentamicin concentrations, which could optimize the gentamicin concentration in lock solutions used in HD catheters.

## 1. Introduction

Central venous catheter-related bloodstream infections (CRBSIs) are one of the most relevant healthcare-associated infections (HAIs) worldwide [1]. In patients hospitalized with acute or chronic kidney disease (CKD), the use of CVC for urgent hemodialysis (HD) therapy and the placement of tunneled catheters are standard procedures. Studies have shown that HD-CRBSISs are mainly caused by Gram-positive bacteria resistant to penicillin and Gram-negative bacteria resistant to cephalosporins [2]. Prevention of CRBSIs involves training healthcare personnel on sterile catheter placement, use of chlorhexidine with alcohol as a skin antiseptic, use of dressings, or use of antiseptic or antibiotic lock solutions on the catheter [1].

Lock solutions involve instilling high concentrations of antiseptic, antibiotic, or combined solutions into the catheter’s lumens. In the case of antibiotic locks, a dose of up to 1000 times its minimum inhibitory concentration (MIC) is used and remains in the catheter for 12 to 24 h to eradicate microorganisms and prevent biofilm formation [3,4].

The lock solutions most frequently used for hemodialysis catheters are heparin, saline solution, ethanol, ethylenediaminetetraacetic acid (EDTA), and trisodium citrate [4], individually or in combination with antibiotics such as cefotaxime, cloxacillin, trimethoprim/sulfamethoxazole, and gentamicin, among others [5].

In recent studies, it has been observed that both citrate and heparin, on their own, do not effectively contribute to the control of catheter infections or prevent the development of bacterial biofilms [6]. However, when these compounds are combined with antibiotics, such as gentamicin (the most widely used), citrate is more effective in preventing catheter-related bloodstream infections than heparin in several studies, even at low gentamicin concentrations [5,7].

Although the clinical results in reducing infections when using gentamicin with citrate/heparin are evident, the antimicrobial effects on bacterial inhibition and biofilm development of these combinations have not been fully described.

The objective of the present study was to analyze and compare the antimicrobial and antibiofilm effects of gentamicin/heparin, gentamicin/citrate, and gentamicin lock solutions on strains of *P. aeruginosa* and *S. aureus* from clinical isolates of catheter-associated infections (CRIs).

## 2. Materials and Methods

### 2.1. Clinical Isolates and Microorganisms

This study used clinical isolates of *S. aureus* and *P. aeruginosa* obtained from blood cultures of hospitalized patients with proven HD-CRBSIs between 2019 and 2023. The cultures were plated on trypticase soy agar (TSA, Condalab^®^, Madrid, Spain) and preserved in trypticase soy broth (TSB, Condalab^®^, Madrid, Spain) with 15% glycerol until the time of use for this study. In addition, the control strains *P. aeruginosa* ATCC 27853 and *S. aureus* ATCC 29213 were used. This work was reviewed and authorized by our institutional research ethics committee under registration code IF23-00005. Although the clinical isolates came from clinical samples, informed consent was not required for this work because it was not a study in humans *per se*.

### 2.2. Antimicrobial Treatment Lock Solutions

Two gentamicin solutions were prepared, one mixed with heparin and the other with sodium citrate. The initial gentamicin concentration for the lock solutions was 8192 µg/mL. For the gentamicin/heparin solution (LSG/H), gentamicin (Sigma–Aldrich, St. Louis, MO, USA) was resuspended in 10 mL of heparin solution (Inhepar^®^ 1000 IU/mL, Laboratorios PISA, Guadalajara, Mexico). To prepare the gentamicin/sodium citrate (LSG/C) lock solution, gentamicin was dissolved together with 0.22 g of sodium citrate dihydrate, 0.073 g of anhydrous citric acid, and 0.245 g of glucose monohydrate in 10 mL of injectable water to have a citrate solution at 30% *w*/*v* at pH 4.5–5.5. Finally, a lock solution containing only gentamicin (LSG) was also used, which was diluted in injectable water.

### 2.3. Biofilm Formation Assay

The biofilm-forming capacity of the bacteria was assessed using the semiquantitative crystal violet assay. Clinical isolates were dissolved in saline with a concentration adjusted to 1.0 on the McFarland scale and diluted 1:100 in trypticase soy broth (TSB). Subsequently, 200 µL of each suspension was transferred into 4 wells of a 96-well polystyrene plate (4 replicates per isolate). In addition, *Stenotrophomonas maltophilia* ATCC 13637 (biofilm-producing control strain) and *Escherichia coli* ATCC 25911 (non-biofilm-producing control strain) were included. After 24 h of incubation at 35 °C, 100 µL of supernatant (corresponding to planktonic cells) was transferred from each strain to new wells and then the optical density (OD) of each well of planktonic cells was measured at 595 nm in a spectrophotometer (Thermo Scientific, Waltham, MA, USA). Subsequently, the remnants of the supernatants were discarded and the wells were allowed to dry in ambient air. The biofilm was fixed with 100 µL of methanol for 10 min, and it was again allowed to dry in ambient air and washed with phosphate-buffered saline (PBS) at pH 7. Next, the biofilm was stained with 100 µL of 0.5% crystal violet for 5 min. Finally, after washing away uncaptured crystal violet, this biofilm was dissolved with 150 µL of 33% glacial acetic acid and shaken at 100 revolutions per minute (rpm) in a 25 °C oscillating incubator for 15 min, and the optical density of each well was measured at 595 nm in the spectrophotometer. Biofilm production was classified according to previously reported criteria [8] based on the difference and relation of the OD.

### 2.4. Minimum Inhibitory Concentration of Planktonic Cells

Antimicrobial susceptibility assays were performed using the broth microdilution method using Mueller–Hinton (MH) broth following the Clinical and Laboratory Standards Institute (CLSI) specifications [9]. The bacterial inoculum used for this assay corresponded to 0.5 on the McFarland scale, and aliquots of 1:150 were prepared and transferred to the 96-well conical bottom plate to obtain an inoculum of ~5.5 CFU/mL Each well was inoculated with the bacterial suspension and 100 µL of gentamicin or lock solutions was added in serial dilutions in triplicate (Table 1). The MIC was determined after incubation via observation until visible growth of the microorganism was not perceived (clear solution) by sight of the same analyst in all cases.

### 2.5. Minimum Biofilm Inhibitory Concentration (MBIC)

The MBIC assay was used to analyze the in vitro effectiveness of gentamicin and lock solutions in preventing biofilm formation. The measurement of biofilm formation was carried out using the crystal violet technique as reported previously [10]. To measure the CMIB, suspensions of the strains at a 1.0 standard concentration of McFarland were diluted 1:100 with MH broth. Then, 100 µL of inoculum was transferred in triplicate to the 96-well plates, as well as 100 µL of gentamicin and the lock solutions in serial dilutions for incubation for 24 h at 35 °C. Subsequently, the planktonic cells were discarded, and the plate was washed with PBS to fix the biofilm with methanol and stained with 0.5% crystal violet for 5 min. Finally, after washing the uncaptured crystal violet, this biofilm was dissolved with 150 µL of glacial acetic acid for 15 min to measure the biofilm formation as mentioned.

### 2.6. Minimum Biofilm Eradication Concentration (MBEC)

Calgary devices (MBEC^®^, Innovotech, Edmonton, AB, Canada) were used to incubate bacterial suspensions of a 1.0 McFarland standard diluted 1:100 in TSB as reported previously [10]. Then, 150 µL was transferred to the wells, ensuring that the lid pins were immersed in the medium, and incubated at 35 °C for 24 h. Subsequently, serial dilutions of gentamicin and the lock solutions with TSB were prepared in a new 96-well flat-bottom plate. The pins were then washed by immersion with PBS to remove non-adhered cells and the Calgary device cap was transferred to the 96-well plate with lock solutions and incubated for 24 h at 35 °C. Afterward, the lid pins were washed and placed in a new 96-well conical bottom plate with TSB, allowed to rest for 30 min, and sonicated for 5 min to release the bacteria and biomass that had adhered to the pegs. Subsequently, the Calgary device cap (MBEC, Innovotech, AB, Canada) was discarded. The plates were incubated aerobically for 24 h at 35 °C. All isolations were performed in triplicate to determine the concentration at which the biofilm of the strain was eradicated (translucent broth).

### 2.7. Hemodialysis Catheter Growth Inhibition Model

For the development of this model, sets of four catheters were used (one for biofilm development and three to evaluate treatments) for each strain that was analyzed. First, the inoculum of the bacterial strains was made at 0.5 of the McFarland standard. Subsequently, dilutions were made to inject the equivalent of approximately 10^6^ colony-forming units (CFU) per mL into the lumens of the MAHURKAR^TM^ 13.5 Fr/Ch catheters (4.5 mm × 19.5 cm dual-lumen, acute dialysis catheters) (1.3 mL). The lumens of the catheters were clamped and incubated for 24 h at 35 °C at 100 rpm (to recreate the body catheter conditions) to observe biofilm development. Nevertheless, we also used special conditions for the enhancement of biofilm growth of *S. aureus*, adding 1% glucose to the inoculum, with incubation at 0 rpm (still conditions) in those catheters.

Subsequently, the contents of the catheters used for biofilm development were emptied aseptically and the microorganisms were recovered. The remaining three catheters were incubated with the lock solutions. The concentrations used were those that demonstrated the biofilm eradication effect in vitro. These catheters were incubated for 72 h at 35 °C (normal residence time of lock solutions in HD patients).

Microorganisms recovered from the catheters were retrieved after the primary endpoints (24 h in biofilm development and 72 h in treatments). For this, the clamps were opened and the contents of the catheters were discarded. With the lumen open, 1 mL of physiological saline solution was injected with a sterile syringe and collected in a sterile tube for the analysis. On the other hand, three segments of 3 cm were cut from the proximal line of the catheter (closest to the patient’s body), introduced into a tube with 3 mL of saline solution, sonicated for 1 min, and mixed by vortex for 15 s. Finally, serial dilutions were prepared to perform cultures on trypticase soy agar plates for the determination of the bacterial count (CFU/mL).

### 2.8. Statistical Analysis

Biofilm formation values were reported as frequencies and percentages depending on the intensity of biofilm formation (mild, moderate, and severe), while MIC, MBIC, and MBEC values were recorded descriptively. Finally, in the analysis of the antimicrobial efficacy of the seal solutions, where the CFU/mL were counted, these were recorded quantitatively, using the mean and standard deviation. Additionally, Student’s *t*-tests were used to compare the CFU/mL of the treatments with the baseline concentration. The *p*-value of <0.05 was considered significant.

## 3. Results

### 3.1. Biofilm Formation of P. aeruginosa and S. aureus

A total of 16 strains were obtained that met all the criteria in the general analysis, of which 11 were *P. aeruginosa* and 5 were *S. aureus*. Two randomly chosen isolates of *P. aeruginosa* (21-0905 and 23-0141) and *P. aeruginosa* ATCC 27853 were analyzed, as well as 2 isolates of *S. aureus* (21-0745 and 23-0140) and *S. aureus* ATCC 29213, to determine the MIC of planktonic cells, MBIC, and MBEC.

**Figure 1:**
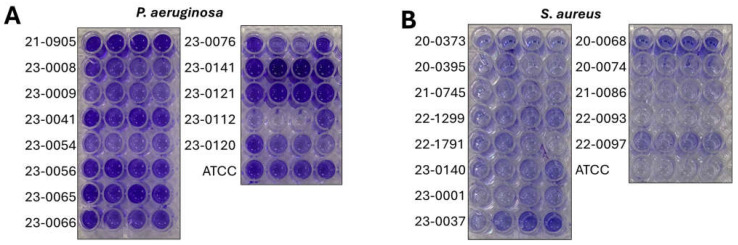
Biofilm production of catheter-isolated bacterial strains. Crystal violet biofilm formation assay in 96-well plates of (**A**) *Pseudomonas aeruginosa* and (**B**) *Staphylococcus aureus*.

All CRIs isolates were biofilm producers. In the *P. aeruginosa* isolates (Figure 1A), 9 (81.98%) showed strong production and 2 (18.2%) showed moderate production. In the case of *S. aureus* (Figure 1B), 1 isolate (20%) was a strong producer, 2 isolates (40%) showed moderate production, and 2 isolates (40%) showed weak production.

### 3.2. Antimicrobial Effect of the Lock Solutions on Planktonic and Biofilm Bacterial Strains

All lock solutions showed better inhibition performances for planktonic cells and biofilm formation than against biofilm eradication (Table 2).

In all the analyzed *S. aureus* strains, the lock solutions showed better effectiveness in inhibiting biofilm cells compared to planktonic cells; however, they were less effective in eradicating cells already established in the biofilm. Interestingly, in clinical isolate 21-0745, considerable atypical resistance to the drug was observed in the locking solutions (Table 2).

This same pattern was repeated in the *P. aeruginosa* strains analyzed in the study, with a slightly better inhibitory effect with LSG/C than with LSG/H (Table 2). However, LSG/H had a better antimicrobial effect for biofilm eradication for both bacterial species than LSG/C (Table 2).

### 3.3. Antimicrobial Effect of Lock Solutions in the Hemodialysis Catheter Biofilm Model

**Figure 2:**
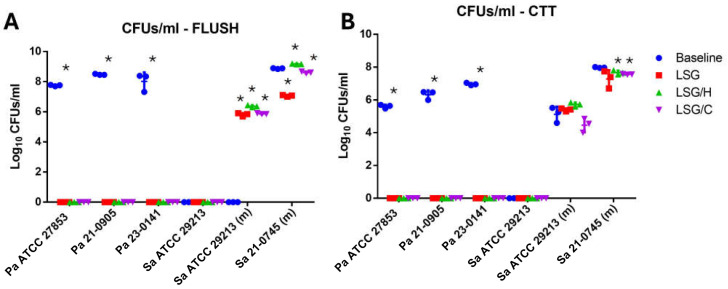
Quantitative antimicrobial effect of lock solutions in the hemodialysis catheter biofilm model: (**A**) colony-forming units quantification of the rinse of untreated (baseline) and treated catheters inoculated with *Pseudomonas aeruginosa* (Pa) and *Staphylococcus aureus* (Sa) isolates; (**B**) colony-forming units per milliliter (CFU/mL) quantification of the suspension from sonicated fragments of the catheter. LSG: lock solution of gentamicin; LSG/H: lock solution of gentamicin/heparin; LSG/C: lock solution of gentamicin/citrate; Pa: *Pseudomonas aeruginosa*; Sa: *Staphylococcus aureus*; (m): modified test conditions; CTT: fragmented and sonicated catheter; * = *p* < 0.05, Student’s *T* test.

In the *P. aeruginosa* strain ATCC 27853 after 24 h of incubation in the catheter, 7.787 log_10_ CFU/mL were obtained on average in the lumen of the catheter, and 5.636 log_10_ CFU/mL in the fragments processed by sonication After treatment with the different lock solutions for 72 h, no colonies were developed in the lumen or catheter fragments (Figure 2). Similar results were observed when analyzing clinical isolates of *P. aeruginosa* 21-0905 and 23-0141.

**Figure 3:**
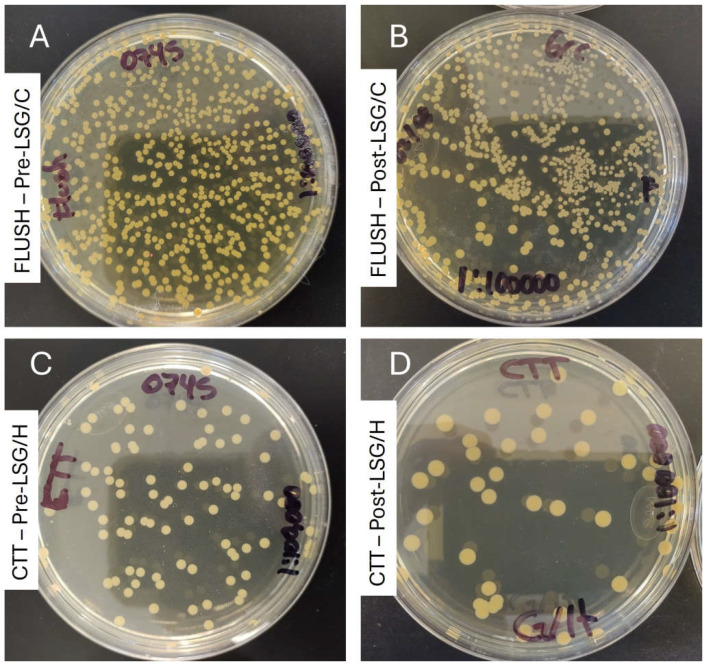
Visual antimicrobial effect of lock solutions in the hemodialysis catheter biofilm model (strain: *S*. *aureus* 23-0745 (gentamicin-resistant)): (**A**) culture of the flush from the untreated catheter at 24 h; (**B**) culture of the flush of the catheter treated with LSG-C for 72 h; (**C**) culture of the suspension from sonicated fragments of the untreated catheter; (**C**,**D**) culture of the suspension from sonicated fragments of the catheter treated with LSG-H for 72 h (all cultures at 1:100,000 for CFU/mL calculations). LSG/H: lock solution of gentamicin/heparin; LSG/C: lock solution of gentamicin/citrate; CTT: fragmented and sonicated catheter.

In the *S. aureus* ATCC 29213 strain, no biofilm formation was observed on the catheters under standard conditions. However, with the modified test conditions, we observed the development of biofilm attached to the catheter fragments (CTT) consisting of an average of 5.12 ± 0.47 log_10_ CFU/mL without the presence of planktonic cells in the flush. After the treatments with the lock solutions, the average CFU/mL in the flush and CTT were increased, except in the CTT with the LSG/C, in which it was slightly reduced (Figure 3A,B). Treatments with the lock solutions showed that the most effective in reducing the bacterial density in the lumen were LSG (8.87 ± 0.028 vs. 7.06 ± 0.058 log_10_ CFU/mL, *p* < 0.0001) and LSG/C (8.87 ± 0.028 vs. 8.60 ± 0.06l log_10_ CFU/mL, *p* = 0.002) (Figure 3C; moreover, treatment with LSG/H significantly increased the bacterial density in the lumen but not in the CTT (Figure 3D).

## 4. Discussion

In our study, all the *S. aureus* and *P. aeruginosa* strains were biofilm producers. Strains recovered from CRIs tend to produce strong biofilms [11,12]. All *S. aureus* isolates analyzed were susceptible to gentamicin (MIC ≤ 2 µg/mL). However, they required higher concentrations for the MIC, MBIC, and MBEC compared to *P. aeruginosa* strains, which could be related to resistance gene acquisition [13]. As in previous reports [14], no correlation was found between the type of biofilm production and the MIC of planktonic cells, MBIC, or MBEC.

All *S. aureus* strains used showed a CLSI-based susceptibility profile for MIC values for both the gentamicin solution and gentamicin/heparin and gentamicin/citrate lock solutions. For *P. aeruginosa* strains, the values were ≤4 μg/mL; however, it is no longer possible to categorize a susceptibility profile for this antibiotic under the latest CLSI update [15]. In our study, the MBIC values of gentamicin for *S. aureus* were 8 μg/mL (except for strain 21-0745) and 4–8 μg/mL for LSG/C, while for *P. aeruginosa* they were 8–32 μg/mL and 4 μg/mL, respectively. The MBEC was 2 to 5 dilutions higher than the MBIC in all cases. To our knowledge, there are no previous reports determining the MIC, MBIC, or MBEC of the seal solutions themselves; such values have only been reported for solutions of antibiotics such as vancomycin, daptomycin, and linezolid without anticoagulant [16,17].

The MBICs of gentamicin/heparin were higher compared to gentamicin/citrate. A previous report showed a significant reduction (1.28 to 0.2 cases/1000 catheter-days) in CRBSI cases caused by *S. aureus*, *P. aeruginosa,* and Enterobacterales after using gentamicin/heparin [18] and gentamicin/citrate [19] lock solutions. Current reports have suggested that citrate lock solutions are generally more effective and safer than heparin lock solutions [7]. Citrate is characterized as a lock solution that has antimicrobial and anticoagulant properties with greater capacities than heparin [20], so its combination with gentamicin could explain its greater antibacterial performance in our study. Nevertheless, regarding its combination with gentamicin, this is the first study to compare both solutions against the same strains in both an in vitro model and a hemodialysis catheter model. Another antimicrobial that has been tested in combination with both heparin and citrate is taurolidine, demonstrating that its citrate formulation is more effective [21]. On the other hand, heparin can act as an inducer of *S. aureus* biofilm formation, reducing and interfering with gentamicin’s antimicrobial effect [22,23]. It is well known that heparin increases the ability of *S. aureus* to develop biofilms on abiotic surfaces through several molecular mechanisms [24]. Additionally, previous reports had described that heparin functions as a substitute for extracellular DNA for the development of the biofilm matrix, and that *S. aureus* possesses heparin-binding molecules [25,26], which could be the reason for this increase in concentration needed to exert an inhibitory antimicrobial effect.

Furthermore, we observed that gentamicin dissolves better in the citrate matrix than in heparin, which provides availability in the medium and enhances the inhibition of bacterial growth; previous studies have also reported this counterproductive interaction between these compounds and their solubility problems [27,28]. However, more strains must be studied to attribute this behavior to heparin in the lock solutions. Additionally, during our inhibition assays, precipitation of the gentamicin/heparin lock solution was observed at the highest concentrations, causing interference in the quantification of biofilm production. Heparin’s biofilm-enhancing effect and its solubility problems with gentamicin represent important disadvantages against it in lock solutions compared to citrate.

Interestingly, in our in vitro assays, LSG/H was superior to LSG/C in terms of biofilm eradication (MBEC). To our knowledge, no previous studies address this specific analysis; however, a much more extensive analysis is needed to demonstrate this apparent superiority of the gentamicin/heparin solution in this area.

All lock solutions at the concentrations tested had a total antimicrobial effect on the biofilm growth of *P. aeruginosa* in the non-tunneled HD catheter model after 72 h of treatment. Previous reports demonstrated a reduction in CRBSIs using a gentamicin/heparin lock solution at 4 mg/mL [18] and depletion of growth using gentamicin (5 mg/mL) with EDTA [29]. Typically, citrate lock solutions contain gentamicin concentrations ranging from 4 mg to 26 mg/mL [7]. In this study, we reported that even using lower concentrations of gentamicin in the lock solutions with heparin (1024 µg/mL) and citrate (2048 µg/mL) is effective in the HD catheter model, setting the standard for reducing and optimizing the concentrations of the lock solutions applied to prevent CRBSIs.

*S. aureus* ATCC 29213 did not cause biofilm formation under the standard conditions of the model [30,31]. To solve this, the strains were incubated without agitation and with 1% glucose in the TSB [32]. With these modifications, *S. aureus* strains can form biofilms and the lock solutions at tested MBECs reduced but failed to eradicate the biofilm formed in the catheter. Ineffectiveness in reducing the bacterial density in catheters could be due to static incubation, as suppressing the fluid dynamics and mechanical agitation reduces the antimicrobial action [16,33].

Although the study’s results show differences in the growth and biofilm development between the different lock solutions analyzed, this study presents a limited number of strains analyzed for MIC, MBIC, and MBEC values, so comparative statistical tests between the administered treatments could not be performed. Therefore, the results are merely descriptive, since *P. aeruginosa* strains showed susceptibility to gentamicin, and strains with strong and weak biofilm production rates were selected, while for *S. aureus* strains, one susceptible strain and one resistant to this antibiotic were selected. Furthermore, in this study, serial dilutions of the stock solutions were used to determine the MIC, MBIC, and MBEC in vitro; therefore, the concentrations of heparin and citrate in the stock solutions decreased with higher dilutions. This could cause coagulation problems when these solutions are tested in patients. However, in the hemodialysis catheter model, the concentrations at which the stock solutions showed efficacy in eradicating biofilms were used, and at these concentrations, the presence of anticoagulant was adequate (heparin 250 IU/citrate 15%). We expect to perform further analyses in the future to determine the role of the antimicrobial effect of the lock solutions used in a clinical setting.

Biofilm inhibition was observed using lock solutions at low gentamicin concentrations compared to lock solutions previously reported and used empirically in clinical practice, raising the possibility of optimizing the concentrations of these solutions for their use in HD catheter-wearing patients.

## Figures and Tables

**Table 1:** Concentrations of gentamicin, heparin, and citrate used in the lock solutions for in vitro MIC, MBIC, and MBEC assays and the hemodialysis catheter model.

LSG	LSG/H		LSG/C	
Gentamicin (µg/mL)	Gentamicin (µg/mL)	Heparin (UI/mL)	Gentamicin (µg/mL)	Citrate (%*w*/*v*)
1024	8192	-	8192	-
512	4096	1000	4096	30.0
256	2048	500	2048 *	15.0 *
128	1024 *	250 *	1024	7.50
64	512	125	512	3.8
32	256	62.5	256	1.9
16	128	31.25	128	0.9
8	64	15.63	64	0.5
4	32	7.81	32	0.2
2	16	3.91	16	0.12
1	8	1.95	8	0.06
0.5	4	0.98	4	0.03
0.25	2	0.49	2	0.01
0.125	1	0.24	1	0.007
0.0625	0.5	0.12	0.5	0.004

Note: µg/mL: micrograms per milliliter; * = concentrations used in the hemodialysis catheter model. Working solutions started at 512 µg/mL, 4096 µg/mL/1000, and 4096 µg/mL/30% for LSG, LSG/H, and LSG/C, respectively.

**Table 2:** Minimal inhibition concentration, minimal biofilm inhibitory concentration, and minimum biofilm eradication concentration values of lock solutions tested on *S. aureus* and *P. aeruginosa* strains.

Strains	LSG (µg/mL)			LSG/H (µg/mL)–(UI/mL)			LSG/C (µg/mL)–(%*w*/*v*)		
	MIC	MBIC	MBEC	MIC	MBIC	MBEC	MIC	MBIC	MBEC
*P. aeruginosa* ATCC 27853	≤0.125	≤0.125	≥256	≤4–1	≥32–8	≥512–125	≤4–0.03	≤4–0.03	≥128–0.9
*P. aeruginosa* 21-0905	≥0.5	≤0.125	≥128	≤4–1	≥16–4	≥64–15.6	≤4–0.03	≤4–0.03	≥1024–7.5
*P. aeruginosa* 23-0141	≥0.5	≥0.25	≥256	≤4–1	≥8–2	≥64–15.6	≤4–0.03	≤4–0.03	≥256–1.9
*S. aureus* ATCC 29213	≤0.125	≤0.125	≥512	≤4–1	≥8–2	≥256–62.5	≤4–0.03	≥8–0.06	≥2048–15
*S. aureus* 21-0745	≥2	≥16	>512	≥64–15.6	>2048–500	>4096–1000	≥1−0.12	≥1024–7.5	>4096–30
*S. aureus* 23-0140	≤0.125	≤0.125	≥16	≤4–1	≥8–2	≥256–62.5	≤4–0.03	≤4–0.03	≥2048–15

Note: µg/mL: micrograms per milliliter; IU/mL: International Units per milliliter; % *w*/*v*: percentage weight–volume.

## Data Availability

The raw data supporting the conclusions of this article will be made available by the authors on request.
